# Altered electrophysiological properties and deranged cardiac autonomic modulation predispose patients to atrial fibrillation after arrested heart operations

**DOI:** 10.1186/1749-8090-8-S1-O62

**Published:** 2013-09-11

**Authors:** JM Kalisnik, A Hrastovec, V Avbelj, B Gersak

**Affiliations:** 1 Department of Cardiovascular Surgery, University Clinical Center Ljubljana, Slovenia; 2 Department of Communications and Computer Networks, Jozef Stefan Institute, Ljubljana, Slovenia

## Background

Advanced Heart Rate (HR) Variability analyses applying non-linear dynamics and chaos theory provide information about derangement of cardiac autonomic modulation predicting ventricular or atrial arrhythmias. Aim of the present study was to determine which high-resolution ECG and non-linear HR dynamics parameters predispose to development of postoperative atrial fibrillation after surgery on the open arrested heart.

## Methods

43 consecutive patients, 26 men, mean age 70.3 yrs referred either for isolated aortic valve replacement ± concomitant coronary revascularization or Bentall procedure were enrolled into the study. High-resolution 20-minute ECG recordings were performed one day before operation to determine RR, PQ, QT and QTc interval as well as non-linear HR parameters by Detrended Fluctuation Analysis (DFA) with short-(≤ 11 beats) and long-term (> 11 beats) correlation properties of R-R intervals. Statistical analyses included paired-samples t-test, Mann-Whitney or Fischer exact test. Results were reported as mean ± SE; p<0.05 or less was considered significant.

## Results

Out of 43 patients 26 developed AF after operation (AF group) and 17 did not (noAF group). The two groups had similar demographic and perioperative characteristics. Cardiopulmonary bypass time (112±28 vs. 97±30 min; p=0.11) and aortic cross-clamp time (83±22 vs. 76±27 min; p=0.15) tended to be longer in AF group. There were no differences in RR, QT or QTc interval between AF and noAF group (64±11 vs. 65±10, 420±32 vs. 436±51 and 432±26 vs. 452±55 ms, respectively; p= NS). DFA parameter α_1_ tended to be higher and DFA α_2_ proved consistently higher in AF group (0.98±0.36 vs. 0.86±0.28; p=0.26 and 0.89±0.17 vs. 0.76±0.18; p=0.018). In addition, PQ interval was consistently shorter (160±20vs. 184±44; p=0.033) in AF group.

## Conclusions

We describe for the first time that patients prone to postoperative AF after arrested heart surgery exhibit profoundly altered non-linear Heart Rate dynamics and shorter PQ interval already preoperatively and independently of perioperative factors. In accordance with the results from our previous beating heart studies, parameter DFA α_2_ comprehensively indicates higher risk of postoperative atrial fibrillation occurence.

